# The Influence of Different Curing Environments on the Mechanical Properties and Reinforcement Mechanism of Dredger Fill Stabilized with Cement and Polypropylene Fibers

**DOI:** 10.3390/ma16216827

**Published:** 2023-10-24

**Authors:** Ying Wang, Chaojie Wang, Zhenhua Hu, Rong Sun

**Affiliations:** 1 College of Transportation, Shandong University of Science and Technology, Qingdao 266590, China; wangying871209@sdust.edu.cn (Y.W.); 202183160020@sdust.edu.cn (C.W.); 2 College of Civil Engineering and Architecture, Shandong University of Science and Technology, Qingdao 266590, China; 13789873609@163.com

**Keywords:** stabilized soil, cement, polypropylene fiber, cohesion, compressive modulus, microstructure

## Abstract

An effective method widely used in geotechnical engineering to solve the shrinkage and cracking issues in cement-stabilized soil (CS) is evenly mixing randomly distributed fibers into it. Dredger fills stabilized with cement and polypropylene fibers (PFCSs) are exposed to rainwater immersion and seawater erosion in coastal areas, influencing their mechanical performance and durability. In this study, direct shear and consolidation compression tests were conducted to investigate the influence of different curing environments on the mechanical properties and compressive behavior of PFCSs. Dominance and regression analyses were used to study the impact of each factor under different curing regimes. The reinforcement mechanism of different curing environments was also explored using scanning electron microscopy (SEM) imaging. The results show that the cohesion and elastic modulus of the specimens cured in seawater were reduced compared with those cured in freshwater and standard curing environments. The best fiber content for the strength and compressive modulus of PFCSs was determined to be 0.9% of the mass of dredged fill. The results of value-added contributions and the relative importance of each factor in different curing environments show that the overall average contribution of cement content in the seawater curing environment is reduced by 6.79% compared to the freshwater environment. Multiple linear regression models were developed, effectively describing the quantitative relationships of different properties under different curing conditions. Further, the shear strength was improved by the coupling effect of soil particles, a C-S-H gel, and polypropylene fibers in the PFCSs. However, the shear strength of the PFCSs was reduced due to the structural damage of the specimens in the freshwater and seawater curing environments.

## 1. Introduction

Coastal reclamation has gained significant attention in recent years. However, dredger fill cannot be directly used for engineering applications due to its high moisture content, high compressibility, and low strength [1]. Therefore, different treatment methods have been proposed to improve the bearing capacity and shear strength of dredger fill. Adding stabilizers to dredger fill improves its mechanical properties [2,3,4,5]. Lime or cement are most commonly used as stabilizing agents. However, various issues associated with cement-stabilized soil (CS) hinder its applicability and prospect. CS can easily undergo shrinkage and cracking when dried due to highly dispersed inorganic colloids in the interior of the CS [6], leading to reduced stability and durability attributes. Compared with other stabilized soils, CS is prone to shedding, fragmentation, and other phenomena. At the same time, CS is inherently brittle under pressure, reducing the bearing capacity after the soil is destroyed [7]. These problems limit the further application and development of CS.

Fiber-reinforced soil is a composite soil in which a certain proportion of fibers are added to the soil to improve its physical and mechanical properties. Some researchers have confirmed that soil strength is improved by uniformly adding fibers because of the high strength and resistance to acid and alkali corrosion. Zhao et al. [8] used polypropylene fibers to stabilize natural sandy soils and studied the effect of different confining pressures, fiber amounts, and fiber lengths on the dynamic characteristics of the resulting fiber-reinforced soil through dynamic triaxial tests. Liu et al. [9] studied the durability of the cotton stalk fiber-reinforced soil and investigated the mechanism of cotton stalk fibers through single fiber tensile and scanning electron microscope tests. Jiang et al. [10] and Zhao et al. [11] studied the effects of fiber length and content on the shear strength behavior of the polypropylene fiber-reinforced soil.

In order to address the various issues associated with CS, several researchers have also attempted to improve CS’s properties by incorporating it with fibers. Tiwari et al. [12], Akbari et al. [13], and Aryal et al. [14] used polypropylene fibers and cement as external additives to stabilize expansive soils, soft soils, and kaolin clays, respectively. It was confirmed that polypropylene fibers significantly increased CS’s strength. Tan et al. [15] used coconut fibers treated by alkali activation and fly ash as external additives and proposed their beneficial use in effectively increasing soil strength and reducing the soil deformation capacity. Qiu et al. [16] quantitatively analyzed the effect of fiber content on soil stabilized by using carbon fibers as an external additive and stabilizing sand through microbial-induced calcite precipitation (MICP). Gobinath et al. [17] found that all the parameters of soil performance were enhanced by using banana fibers as an additive to improve soil stabilized by sodium carbonate. Cao et al. [18] studied the unconfined compressive strength (UCS) and microstructure of soils stabilized by basalt fibers, cement, and fly ash. Similarly, Hu et al. [19] evaluated the reinforcement mechanism of CS and alginate fibers and developed a compressive strength correction prediction model considering multiple parameters. In addition, Zhang et al. [20] used recycled GFRP (glass fiber-reinforced polymer) fibers and cement as additives and showed that the UCS of stabilized soil significantly increased when the fiber content was 5% of the wet soil mass.

In summary, improving the mechanical properties of dredger fill stabilized with cement and polypropylene fibers (PFCSs) is feasible. However, many factors can affect the resulting mechanical properties of PFCSs, especially when PFCSs are used in coastal areas where factors like precipitation and seawater erosion can influence their solidification. Hence, it is imperative to clarify the effect of different curing environments on the mechanical properties of PFCSs with various parameters before utilizing them for resource utilization. Based on these considerations, the effects of moisture content, polypropylene fiber content, cement content, and curing age on the shear strength and compression characteristics of PFCSs in different curing environments were studied in this paper through direct shear and consolidation compression tests. Furthermore, a dominance analysis was applied to analyze the impact of each factor on cohesion in different curing environments, and a regression analysis was used to establish linear equations between each factor and cohesion in different curing environments. Furthermore, SEM tests were conducted to analyze the reinforcement mechanism of PFCSs under different curing environments. These measures provide a scientific basis for resource utilization.

## 2. Raw Materials and Experimental Methods

### 2.1. Raw Materials

**Figure 1:**
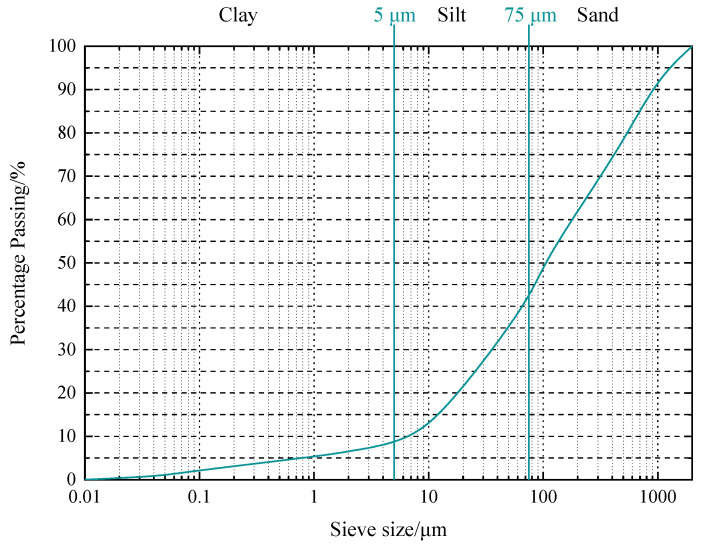
Grain size distribution curve of dredger fill.

The soil used in this experiment was derived from the dredger fill of Xingguang Island in Qingdao. The basic physical properties of the dredger fill were determined following ASTM D854 [21], ASTM D4318 [22], ASTM D2216 [23], ASTM D7263 [24], and ASTM D2487 [25]. The basic physical characteristics of the dredger fill are listed in Table 1, while the particle size distribution (gradation) curve is shown in Figure 1.

In this study, polypropylene fibers and cement were used as stabilized agents to treat the dredger fill. The physical properties of the polypropylene fibers obtained from the manufacturer are given in Table 2. Cement-type PC 42.5 used in this study was produced by Anhui Conch Cement Co., Ltd., located in Anhui Province of China. The chemical composition of cement is presented in Table 3.

### 2.2. Sample Preparation

**Figure 2:**
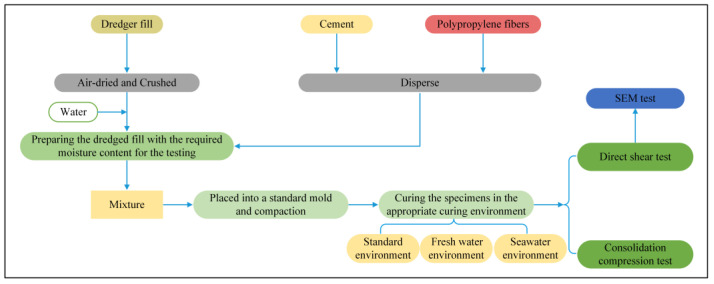
The experimental procedure.

The experimental procedure is shown in Figure 2. First, the specimens were prepared according to the experimental procedure, followed by subjecting direct shear and consolidation compression tests. Finally, SEM imaging was performed on the specimens. Comprehensive details on the specific procedures and conditions used in these tests are provided in a later section.

### 2.3. Experimental Method

Direct shear tests were carried out to obtain the strength and deformation characteristics of PFCSs in this study. In addition, one-dimensional consolidation tests were used to analyze the mechanical properties of PFCSs. SEM imaging was conducted to study the strength and deformation characteristics.

In order to obtain the shear strength characteristics of PFCSs, the direct shear test was conducted using a ZJ-type strain-controlled direct shear apparatus, following ASTM D3080 [26]. The specimens of the shear tests were subjected to three vertical pressures (100 kPa, 200 kPa, and 300 kPa). In this investigation, three samples were prepared for each mix, and the average was taken as the representative value. The average error was also obtained following each test. The test results were considered suitable if the average error was <5%. The specific test cases are listed in Table 4.

In order to analyze the compressive behavior of PFCSs, a consolidation compression test was carried out using a WG-type triaxial high-pressure single-lever consolidation apparatus based on ASTM D2435 [27]. During the consolidation compression test, loading was applied under 12.5, 25, 50, 100, 200, 300, 400, 800, and 1600 kPa. Each load level was kept stable for 24 h, with the test lasting 9 days. In this investigation, three samples were prepared from each mix. A series of cement contents (6% by weight of the wet soil), fiber contents (0.6%, 0.9%, or 1.2% by weight of the wet soil), a curing duration (7 days), and the curing environments (freshwater and seawater) were considered with a constant water content (1.0 W_L_) of the dredger fill in this study.

In order to study the mechanism of the changes in the mechanical properties of PFCSs from a microscopic perspective, SEM testing was conducted using the Nova NanoSEM450 scanning electron microscope instrument manufactured by FEI Company in Hillsboro, OR, USA. Specimens for SEM testing were taken from obvious locations of the failed sample, and the specimen size was controlled to 5 mm × 5 mm × 5 mm to ensure consistency. The specimen was soaked in anhydrous ethanol under vacuum saturation for 48 h. Subsequently, the specimen was placed in a vacuum-drying oven at a suitable temperature to avoid damage to the specimen surface. Finally, the dried specimen was stored in a dry and well-ventilated place.

Secondary processing was performed on the specimen to control its size to 3 mm × 3 mm × 2 mm. The processed specimen was placed inside a vacuum sputter coater machine for gold coating. Then, the processed specimen with a thin gold film was transferred into the sample chamber of the SEM. The sample was observed under the microscope to observe its microstructure. An appropriate magnification was chosen during the observation to show the sample’s microstructure adequately. An electron beam focused on the same spot for too long was avoided to prevent damaging the sample.

## 3. Results, Analysis, and Discussion

### 3.1. Mechanical Properties of PFCSs

In order to characterize PFCSs’ shear strength, the cohesion and friction angle were obtained through direct shear tests [28,29]. In these tests, the change in the friction angle of each sample was insignificant, mainly concentrated between 10° and 20°. Hence, only the cohesion was analyzed in this paper in detail.

#### 3.1.1. Cohesion of Different Specimens

**Figure 3:**
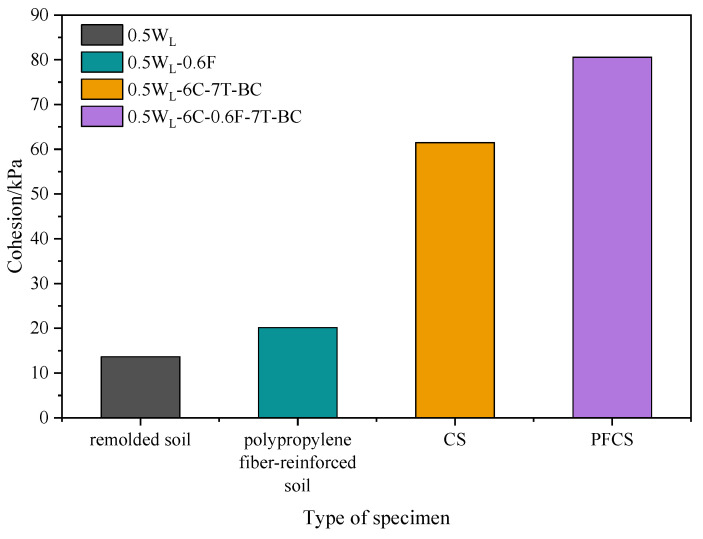
Cohesion of different specimens.

The influence of different materials on the cohesion could be obtained by comparing the cohesion of different samples to carry out relevant research in the later stage. As shown in Figure 3, the highest cohesion was with PFCSs, the second one was with CS, and the remolded soil had the least cohesion. The cohesion of the polypropylene fiber-reinforced soil increased by 47.55% compared with that of the remolded soil. Also, the cohesion of the CS increased by 350.40% compared with that of the remolded soil. The results indicate that soil cohesion was improved by adding polypropylene fibers or cement, and the effect of cement on cohesion improvement was higher than that of polypropylene fibers. Further, PFCS cohesion increased by 300.00% compared with the soil reinforced with polypropylene fibers and increased by 31.03% compared with the CS. Therefore, it can be inferred that the soil cohesion was improved more effectively by adding polypropylene fibers and cement.

#### 3.1.2. Cohesion of Different Curing Environments

**Figure 4:**
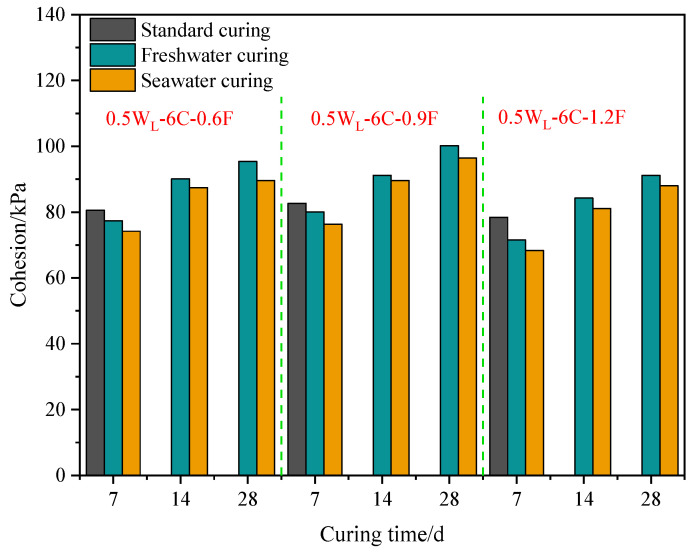
Cohesion of specimens with different curing environments.

The curing environment can alter the physical and chemical reactions in PFCSs, significantly impacting its strength. Figure 4 shows that the cohesion in the standard curing environment was highest, followed by that in the freshwater curing environment, while the lowest cohesion was observed for the specimens cured in seawater. Taking a polypropylene fiber content of 0.6% as an example, the cohesion of samples with a curing time of 7 d decreased by 3.95% in the freshwater curing environment and by 4.11% in the seawater curing environment, compared to that of samples cured under standard conditions. At 14 d and 28 d curing ages, the cohesion in the seawater curing environment decreased by 2.94% and 6.11%, respectively, compared with that in the freshwater curing environment. Similarly, the same declining pattern was exhibited for other polypropylene fiber contents. However, a polypropylene fiber content of 1.2% had a better effect.

The reduced cohesion observed in the specimens is because the soil had water absorption and swelling characteristics, and irregular deformation occurred when the sample was immersed in water. When this deformation exceeded the limit of the internal structure of the soil, microcracks appeared due to the stress concentration [1], damaging the internal structure of the soil. In the case of samples cured in a seawater environment, the main reason for the decrease in strength was attributed to the interference of seawater on the hydration reaction. Soaking any cementitious material in seawater causes the precipitation of calcium elements and Friedel’s salt [30], destroying the C-S-H gel structure and generating pores in the sample.

#### 3.1.3. Cohesion of Different Polypropylene Fiber Contents

**Figure 5:**
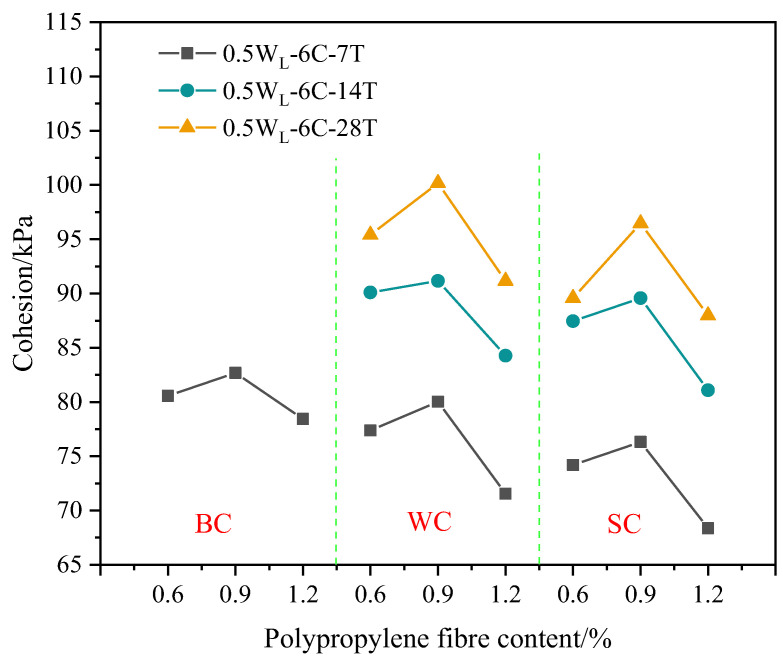
Cohesion of specimens with different polypropylene fiber contents.

Fiber content has a critical role in the enhancement of the mechanical behavior of composites. Figure 5 shows that the cohesion of the sample in a freshwater curing environment with a polypropylene fiber content of 0.9% increased by 3.42% compared with that of the sample with a polypropylene fiber content of 0.6% at a 7 d curing age. However, the cohesion strength of the sample with a polypropylene fiber content of 1.2% decreased by 10.60% compared to that of the sample with a polypropylene fiber content of 0.9%. Similarly, the same pattern of change was exhibited in both standard and seawater curing environments. This explains why PFCS cohesion first increased and then decreased with the increasing fiber content. Further, the sample with a polypropylene fiber content of 0.9% had the largest cohesion strength under different curing conditions.

This phenomenon is because the mechanism of polypropylene fibers was similar to that of plant roots reinforcing soil [31]. Based on this mechanism, it can be known that the CS pores were filled by polypropylene fibers, forming a denser structure of the PFCSs. Meanwhile, frictional force was generated by the contact area between the polypropylene fiber and the mixed material particles based on friction theory when the PFCSs were subject to a lateral load, which weakened the external load and suppressed the soil deformation [15]. However, when the polypropylene fiber content was greater than 0.9%, a negative effect on the improvement of the cohesion of the PFCSs appeared by further increasing the polypropylene fiber content. A large amount of polypropylene fibers were difficult to distribute evenly in the PFCSs, so fibers tended to form clusters and entangled with each other, damaging the overall structure of the soil [31]. Nevertheless, the main reason for the increase in cohesion of the CS was the production of C-S-H colloids [32], which could improve the overall integrity and performance of the soil. However, the too-high polypropylene fiber content could reduce the binding ability of cement, thus reducing the inter-particle interaction forces in the soil [33]. As shown in Figure 3, cement had a higher effect on improving cohesion than polypropylene fibers, which can macroscopically lead to a decreased cohesion of the PFCSs. This also explains the increase of the reduced ratio in the freshwater and seawater environments with a fiber content of 1.2%, as shown in Figure 4.

#### 3.1.4. Cohesion Due to Varying Cement Contents

**Figure 6:**
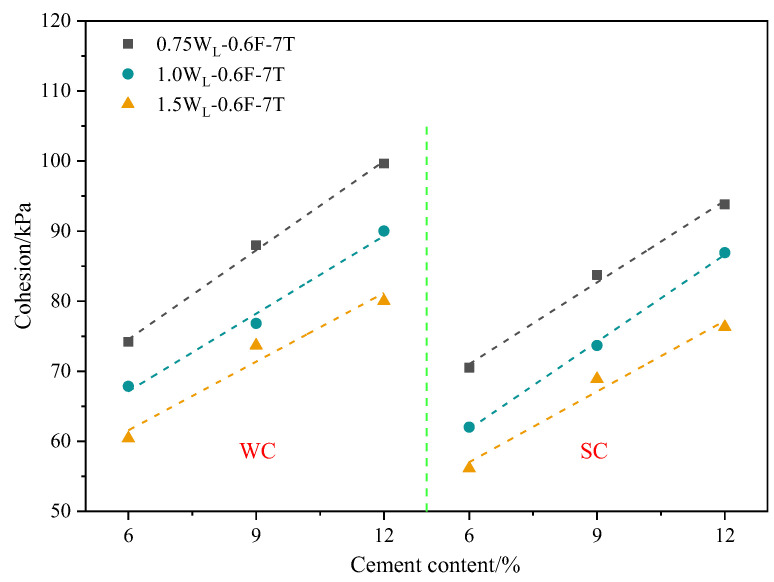
Cohesion of specimens with different cement contents.

Figure 6 shows that the cohesion of the sample in the freshwater curing environment linearly increased with the cement content. It also shows that the cohesion increased linearly with the cement content under seawater curing.

The main reason for this was that the C-S-H gel produced by the hydration reaction of cement possessed a bonding effect, which bonded together the soil particles in the PFCSs [33]. Since the C-S-H gel structure is smaller than the soil particles, the pores of the particles were filled in the soil; hence, the soil structure was more compact, and finally, denser units were formed [33]. Also, the structure of the soil was more robust after the production of cement hydration products due to the higher strength of the C-S-H gel after hardening [34].

#### 3.1.5. Cohesion of Dredger Fill with Varying Moisture Contents

**Figure 7:**
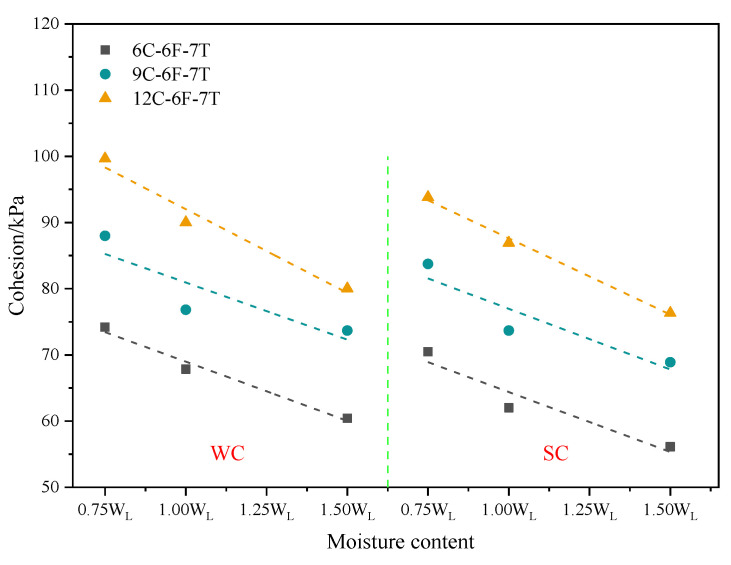
Cohesion of specimens with different moisture contents.

As shown in Figure 7, the cohesion of the sample in the freshwater curing environment with a moisture content of 1.0 W_L_ decreased by 8.57%, 12.67%, and 9.66%, respectively, compared with that of the sample with a moisture content of 0.75 W_L_ when cement contents were 6%, 9%, and 12%. The cohesion of the sample with a moisture content of 1.5 WL decreased by 10.94%, 4.11%, and 11.09%, respectively, compared with that of the sample with a moisture content of 1.0 W_L_. Through the data, it is found that the cohesion decreased with the increase of the moisture content of the soil in the freshwater curing environment. This also reflects that the cohesion of the sample decreased with the increasing moisture content of the dredger fill in the seawater curing environments.

The internal interaction force between soil particles was van der Waals force [1], mainly composed of the molecular attraction of bonding material and bound water film. With the continuously increasing moisture content, the number of pores in the soil gradually increased, weakening the bonding effect between soil particles. Consequently, the van der Waals force decreased in the soil. In addition, a certain lubricating effect of water itself was possessed [30], and the increase in water content would expand this effect, making it easier for the soil to experience relative sliding under a lateral shear force.

#### 3.1.6. Cohesion under Varying Curing Times

**Figure 8:**
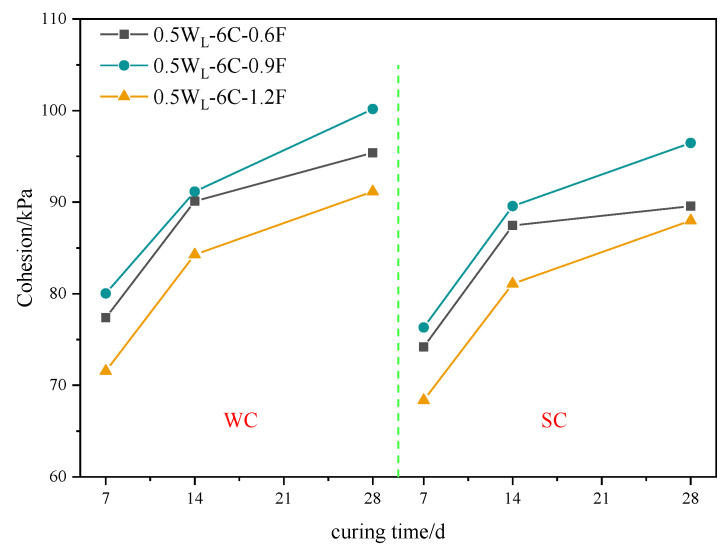
Cohesion of specimens with different curing times.

Figure 8 shows the cohesion results under varying curing times. When the polypropylene fiber contents were 0.6%, 0.9%, and 1.2%, the cohesion in the freshwater curing environment with a curing age of 14 d increased by 16.44%, 13.91%, and 17.78%, respectively, compared to that with a 7 d curing age. The cohesion with a curing time of 28 d increased by 5.88%, 9.88%, and 8.18%, respectively, compared to that with a curing time of 14 d. Similarly, the same pattern was exhibited in the seawater curing environment. It is also observed that the increasing cohesion with a curing time from 7 d to 14 d was greater than that from 14 d to 28 d, which is consistent with the findings of Deng et al. [35]. This is because the cohesion of PFCSs was affected by the curing time due to an increased cement hydration reaction. As the curing time increased, cement hydration further progressed. Although the C-S-H gel continued to be produced, the yield gradually decreased, resulting in a smaller improvement in the cohesion strength of the sample.

### 3.2. Compressive Properties of PFCSs

Foundation settlement is an important indicator for describing foundation deformation. When the stabilized soil of the dredger fill is directly used as a filling material, it is imperative to obtain the compression indicators of the stabilized soil to evaluate the foundation settlement. Therefore, the compression indicators of PFCSs should be calculated first before practical engineering applications.

#### 3.2.1. Compression Curves of PFCSs

**Figure 9:**
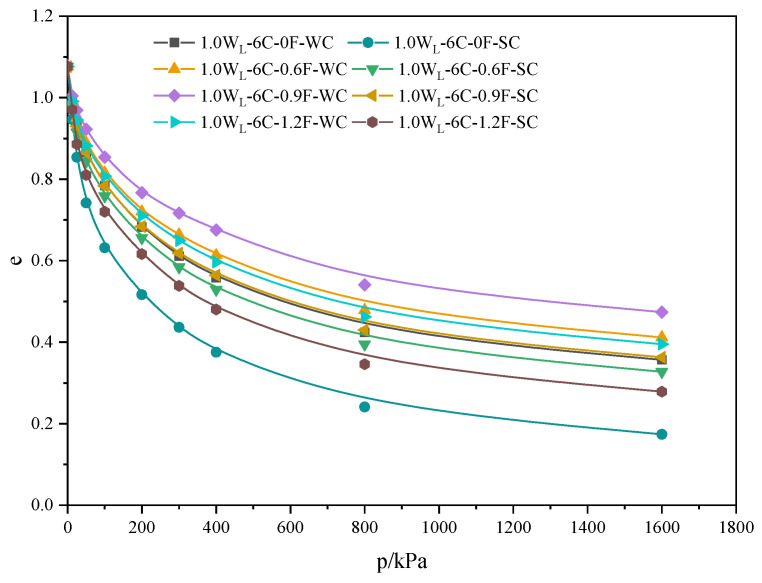
Compression curves of different specimens.

Figure 9 shows that the specimen with a polypropylene fiber content of 0.9% exhibited optimal compressive properties in freshwater and seawater curing environments. The specimen without polypropylene fiber had the poorest compressive properties. The decrease in the void ratio with PFCSs was mainly due to the spatial network structure and bridging effect [36] formed by the polypropylene fibers inside the sample. Further, the sample with a polypropylene fiber content of 0.9% had the best reinforcement effect, validating the results in Figure 5. Also, the void ratio of specimens cured in a freshwater environment was higher than those cured in a seawater environment under the same conditions. This might be because the hydration reaction can be interfered with and damaged in the seawater environment, leading to erosion and the swelling effect of dredger fill’s water absorption. This is consistent with the results presented in Figure 4.

#### 3.2.2. Compressive Modulus of PFCSs

**Figure 10:**
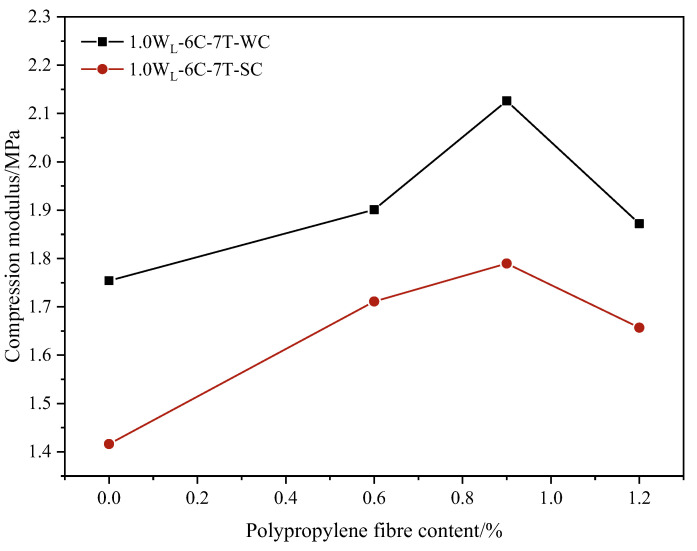
Compressive modulus of samples with different polypropylene fiber contents.

As shown in Figure 10, the compressive modulus increased initially and then decreased with the increasing content of fibers. The largest compressive modulus of specimens was obtained for 0.9% of polypropylene fiber content, while the smallest compressive modulus was observed for the samples without any polypropylene fiber content. Taking the freshwater curing environment as an example, the compressive modulus of samples with 0.9% of polypropylene fiber content increased by 21.21% than those without any polypropylene fiber content. This result further confirms that the compressive modulus was reduced by adding polypropylene fibers into CS and proved that the best reinforcement effect of the CS improved by fibers could be provided with 0.9% of polypropylene fiber content.

Meanwhile, it can be observed that under the same conditions in Figure 10, the compressive modulus of the samples cured in a freshwater environment was higher than that of the samples cured in a seawater environment. When the polypropylene fiber content was 0.9%, the compressive modulus of the samples cured in a freshwater environment increased by 18.81% compared to those cured in a seawater environment. This result can further validate the findings given earlier in Figure 4.

### 3.3. Analysis and Discussion

1. Comparing the cohesion of four soils, the highest cohesion was found for PFCSs, followed by CS, while the remolded soil had the lowest cohesion strength. The soil cohesion improved more effectively by synergistically adding polypropylene fibers and cement.
2. For different curing environments, the cohesion of the specimens cured in a standard curing environment was improved by 4.11% compared with those cured in a freshwater curing environment, while those cured in a freshwater curing environment were improved by 3.03–6.51% compared with those cured in a seawater curing environment, and the compressive modulus of the specimens cured in a seawater curing environment was reduced by 18.81% compared to that of the samples cured in a freshwater environment.
3. The mechanical properties and compressive behavior of PFCSs were studied under different curing environments. The cohesion was improved by adding polypropylene fibers into CS, and the compressibility was also reduced effectively. The best polypropylene fiber content was determined to be 0.9%. The cohesion of PFCSs increased with the increasing cement content and curing time but decreased with the increase of moisture content.

## 4. Experimental Data Analysis

### 4.1. The Dominance Analysis Method 

#### 4.1.1. Correlation Analysis

In order to conduct a correlation analysis on the cohesion of PFCSs, four influencing factors were selected, i.e., moisture content, polypropylene fiber content, cement dosage, and curing time. In a freshwater curing environment, the correlation coefficients between the four factors and cohesion were 0.494, 0.104, 0.281, and 0.623, whereas, under seawater curing, the correlation coefficients between the four factors and cohesion were 0.521, 0.109, 0.258, and 0.616. From the correlation coefficients, it is observed that the four influencing factors have an impact on cohesive strength. Among them, the curing time has the highest influence on cohesion, while the polypropylene fiber content has the least impact.

#### 4.1.2. Qualitative Analysis of Advantages

The dominance analysis method was used to assess the impact of each factor on cohesion. The dominance relationship in the dominance analysis method can be divided into complete dominance and general dominance levels. Complete dominance is the condition in which the relative importance sequence of each predictor variable is constant across all sub-models. General dominance refers to the relative importance sequence of predictor variables under the overall average contribution.

Four influencing factors (moisture content, X_1_; polypropylene fiber content, X_2_; cement content, X_3_; and curing time, X_4_) on cohesion as the dependent variable were selected, and the changes in the correlation coefficients were calculated when each influencing factor was included in separate sub-models without considering its influence. The change in correlation coefficient could represent the contribution of each influencing factor in terms of value added. The average value of the value-added contribution also represented the advantage weight (average contribution) of these influencing factors. The relation for calculating is given as Equation (1).

(1) $$C_{X_{i}}^{\left(k\right)}=(\sum R_{yX_{h}X_{i}}^{2})/(k^{P}-1)$$

where $C_{X_{i}}^{\left(k\right)}$ is the average contribution of a variable when k influencing factors are contained in a sub-model, and this average contribution is with respect to the dependent variable y; $R_{yx_{h}x_{i}}^{2}$ is the change in correlation coefficient when a variable is added to a sub-model with k influencing factors; $X_{h}$ is the k influencing factors; P is the number of influencing factors; and k is the number of influencing factors (k = 0, …, P − 1).

The relation for the total average contribution of influencing factors on cohesion is given by Equation (2).

(2) $$C_{X_{i}}=\frac{1}{P}\sum_{k=0}^{P-1}C_{X_{i}}^{\left(k\right)}$$

The magnitude of the total average contribution of each influencing factor could reflect its respective importance. According to Equations (1) and (2), the incremental contribution and total average contribution of the four influencing factors (moisture content, X_1_; polypropylene fiber contents, X_2_; cement contents, X_3_; and curing time X_4_) on cohesion were calculated in the freshwater curing environment. The results are listed in Table 5.

In a freshwater curing environment, the magnitude order of the four factors influencing the cohesion of the specimens was curing time > cement content > water content > polypropylene fiber content.

From the complete dominance and general dominance perspectives, the value-added contributions for six pairs of variables (X_1_ versus X_2_, X_1_ versus X_3_, X_1_ versus X_4_, X_2_ versus X_3_, X_2_ versus X_4_, and X_3_ versus X_4_) were compared by analyzing the data in Table 5. The results with non-empty value-added contributions were produced as follows: X_1_ had a complete advantage over X_2_, X_4_ had a complete advantage over X_1_, X_3_ had a complete advantage over X_2_, X_4_ had a complete advantage over X_2_, X_3_ had a general advantage over X_1_, and X_4_ had a general advantage over X_3_.

Based on Equations (1) and (2), the calculation of the incremental and total average contributions of four influencing factors (moisture content, X_1_; polypropylene fiber content, X_2_; cement content, X_3_; and curing time, X_4_) on cohesion in the seawater curing environment are presented in Table 6.

For the perspective of relative importance in Table 6, it can be observed that in the seawater curing environment, the order of the magnitude of the impact of the four factors on cohesion was curing time > moisture content > cement content > polypropylene fiber content. Compared with the results obtained for the freshwater curing environment, the impact of cement content was reduced under the seawater curing environment.

Similarly, the comparison results with non-empty value-added contributions from complete dominance and general dominance perspectives were represented as follows: X_1_ had a complete advantage over X_2_; X_3_ had a complete advantage over X_2_; X_4_ had a complete advantage over X_2_; X_1_ had a general advantage over X_3_; X_4_ had a general advantage over X_1_; and X_4_ had a general advantage over X_3_. Compared with the results of value-added contributions in the freshwater curing environment, the opposing trend was reflected in the value-added contributions for X_1_ and X_3_, indicating that the effect of cement content was reduced. The comparison results were consistent with the results of relative importance.

The influence of cement content was reduced in the seawater curing environment compared with that in the freshwater curing environment. This is because the main chemical composition of ordinary Portland cement in the seawater environments has ${\mathrm{CaO},\;\mathrm{SiO}}_{2}{,\;\mathrm{Al}}_{2}O_{3}{,\;\mathrm{Fe}}_{2}O_{3}{,\;\mathrm{and}\;\mathrm{SO}}_{2}$. The main ions eroded by seawater are ${\mathrm{Mg}}^{2+}{,\;\mathrm{Cl}}^{-}{,\;\mathrm{and}\;\mathrm{SO}}_{4}^{2-}$. First, ${\mathrm{Mg}}^{2+}$ ions reacted with cement soil to form $MgO\cdot SiO_{2}\cdot H_{2}O$, and $3\mathrm{CaO}\cdot SiO_{2}\cdot 2H_{2}O$ was dispersed, reducing the cementitious properties, thus lowering the strength of the stabilized soil.

### 4.2. Regression Analysis

#### 4.2.1. Mathematical Modeling

A regression analysis was conducted to establish the quantitative relationship between cohesion and the influencing factors to analyze the influence of various factors on the cohesion of PFCSs in freshwater and seawater curing environments. Hence, a multiple linear model was developed among the cohesion, moisture content, polypropylene fiber content, cement content, and curing time.

Based on the experimental data, a multiple linear regression analysis was conducted on the test results using IBM SPSS Statistics for Windows, Version 27.0 (IBM Corp., Armonk, NY, USA). The results are listed in Table 7, Table 8 and Table 9.

Based on the coefficients in Table 9, the four-variable linear regression equation for the cohesion of the test samples in the freshwater curing environment was expressed as Equation (3).

(3) c=60.202−0.348W−3.469F+4.018C+0.920T

where c is cohesion (kPa); W is the moisture content (%); F is the polypropylene fiber content (%); C is the cement content (%); and T is the curing time (d).

In order to better quantify the impact of freshwater and seawater curing environments on the cohesion of PFCSs, a reduction formula is defined as given in Equation (4).

(4) $$\eta =\frac{c_{s}}{c_{w}}$$

where η is the reduction coefficient; $c_{s}$ is the cohesion of the samples in the seawater curing environment (kPa); and $c_{w}$ is the cohesion of the samples in the freshwater curing environment (kPa).

The cohesion of the soil samples under both curing environments was studied by analyzing experimental data to calculate the reduction coefficient. The average of these reduction coefficients was taken as the overall reduction coefficient in this study. Thus, the overall reduction coefficient was determined to be 0.953 from Equation (4). The four-variable linear regression equation for the cohesion of the samples in the seawater curing environment with various influencing factors is given in Equation (5).

(5) $$c=0.953\times \left(60.202-0.348W-3.469F+4.018C+0.920T\right)$$

#### 4.2.2. Model Validation

The experimental results were validated to corroborate the reliability of the regression equation. The experimental values and predicted values of the cohesion of the samples in the freshwater and seawater curing environments are presented in Table 10 and Table 11.

In regression analysis, the error ratio and small error probability are important indicators for testing the model accuracy. The calculation equation is given as Equation (6).

(6) $$C=\frac{S_{2}}{S_{1}}$$

(7) $$P=\left\{\left|\left(\varepsilon^{\left(0\right)}(t)-{\bar{\varepsilon }}^{\left(0\right)}\right)\right|\right.\left.<0.6745S_{1}\right\}$$

where C is the error ratio; $S_{1}$ is the mean squared deviation of the observed values; $S_{2}$ is the mean squared deviation of the residual sequence; P is the small error probability; $\varepsilon^{\left(0\right)}(t)$ is the residual values (kPa); and ${\bar{\varepsilon }}^{\left(0\right)}$ is the mean of the residuals (kPa).

The grey forecasting accuracy test criteria are presented in Table 12.

It is found that the coefficient C in Equation (3) is 0.31 and P is 1.00 by calculating using Equations (6) and (7). For Equation (5), the coefficient C is 0.34 and P is 0.95. This indicates that Equations (3) and (5) can effectively describe the quantitative relationship between the cohesion and moisture content, polypropylene fiber content, cement content, and curing time in freshwater and seawater curing environments.

## 5. Microscopic Mechanism of PFCSs

**Figure 11:**
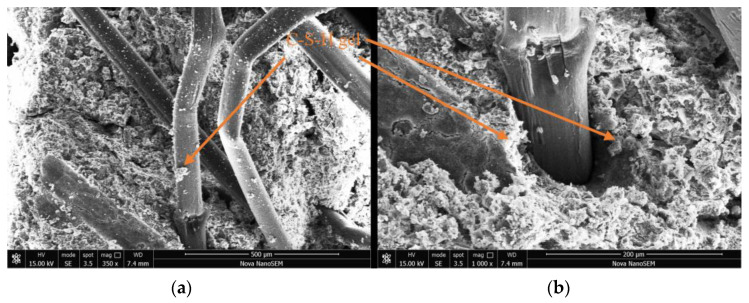
SEM images of PFCSs: (**a**) ×350, (**b**) ×1000.

The polypropylene fibers were randomly distributed in the CS, forming a spatial mesh structure, as shown in Figure 11. Since fibers cannot participate in cement hydration reactions, the relative frictional force provided by the rough surface of the fiber enhanced the PFCSs’ cohesion [36,37]. Further, the polypropylene fibers limit particle movement and suppress cracks effectively [36,38]. In addition, a larger surface area for attaching to the C-S-H gel and soil particles was generated in the fiber with a rough surface, compensating for density reduction caused by fiber bonding. Inside the PFCSs, a mutual coupling effect existed between soil particles, the C-S-H gel, and polypropylene fibers—C-S-H gel-bonded soil particles and polypropylene fibers together, allowing for loose soil to aggregate and increasing internal stability and reducing its deformation capacity. Meanwhile, the C-S-H gel produced by the hydration reaction covered the fiber surface, making their surface rougher and increasing the frictional force between the fibers and soil particles. This significantly enhanced the ability of the polypropylene fibers to capture soil particles. Finally, a more solid overall structure inside the soil was formed by connecting the larger soil particles with better bonding to the less cohesive soil particles, reducing the deformation in the stabilized soil.

**Figure 12:**
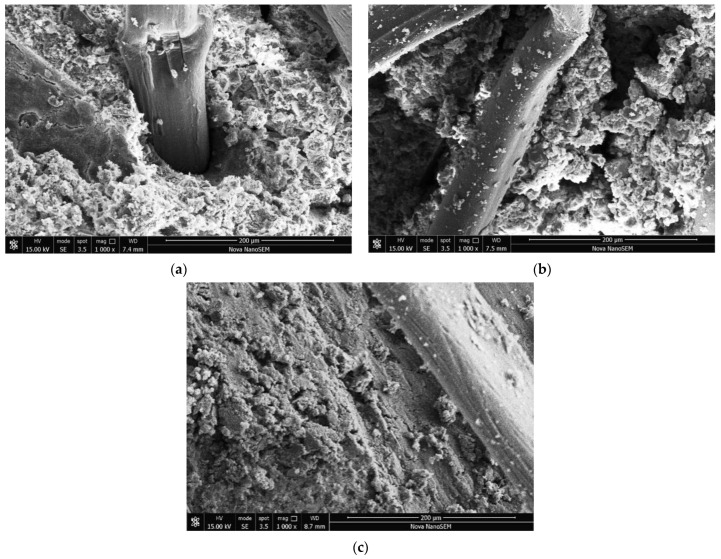
SEM images of PFCSs with different curing environments: (**a**) Standard curing environment. (**b**) Freshwater curing environment. (**c**) Seawater curing environment.

Figure 12a,b show that the larger volumes of mixed particles comprising soil particles and the C-S-H gel were exhibited in the specimens cured in the freshwater curing environment compared with the specimens in the standard curing environment, so a greater C-S-H gel was required to encapsulate and connect these aggregates. In this case, some aggregates could not be adequately connected to neighboring aggregates, resulting in weak internal structures within the specimens. Hence, the failure and deformation of specimens may occur due to these weak structures. Additionally, this shows that a denser structure can be produced by closely connecting the aggregates on the surface of specimens in the standard curing environment. In contrast, noticeable cracks between the aggregates were exhibited in specimens cured in a freshwater curing environment. These cracks reduced the ability of PFCSs to resist deformation. As shown in Figure 12c, it can be proposed that the relatively smooth C-S-H gel with only a small amount of distribution appeared in seawater curing environments compared with the specimens cured in standard and freshwater environments, and the specimens in seawater curing environments showed a higher presence of densely distributed microcracks internally. In addition, the main reason for the increased strength of the PFCSs was the formation of a C-S-H gel [33]. However, the overall density within the PFCSs in seawater curing environments was reduced due to the decrease in the number of C-S-H-gel formations and microcracks, which decreased the mechanical performance.

## 6. Conclusions

In this study, the mechanical and compressive properties of PFCSs were analyzed through direct shear and consolidation compression tests with different curing environments. The impact of each factor in the different curing environments was analyzed by the dominance analysis and regression analysis, and the microscopic mechanism was explored by SEM imaging. The main conclusions drawn from the obtained results are as follows.

1. The improvement of soil cohesion by adding polypropylene fibers and cement was more pronounced. The cohesion of the specimens cured under the standard curing environment was improved by 4.11% compared with those cured in the freshwater curing environment, whereas those cured in the freshwater curing environment were improved by 3.03–6.51% compared with those cured in the seawater curing environment. PFCS cohesion increased with the cement content and curing time but decreased with the increasing moisture content. The compression behavior of PFCSs was analyzed through consolidation testing, and the compressive modulus of the specimens cured in the seawater curing environment was reduced by 18.81% compared to those cured in the freshwater environment. The best polypropylene fiber content in direct shear and consolidation specimens was 0.9%.
2. The impact of each factor on cohesion was analyzed by the dominance analysis method. Comparing the results of value-added contributions and the relative importance of the specimens in freshwater and seawater curing environments, the results indicate that the impact of cement content had been reduced in the seawater curing environment. In addition, multiple linear regression models were established in freshwater and seawater curing environments, and the models can effectively describe the quantitative relationship between the cohesion and moisture content, polypropylene fiber content, cement content, and curing time in both freshwater and seawater curing environments.
3. A unique network structure formed by adding polypropylene fibers into CS was observed through SEM tests. The synergistic action with the C-S-H gel, soil particles, and polypropylene fibers improved the overall structural stability of the PFCSs. Compared to the standard-cured specimens, the larger aggregate volumes and noticeable cracks of freshwater-cured specimens were produced at the aggregate–binder interface. The amount of C-S-H gel formed was significantly lower in seawater-cured specimens than in the standard-cured and freshwater-cured specimens. The microscopic structural characteristics contribute to the decreased mechanical properties of PFCSs.

## Figures and Tables

**Table 1:** Basic physical characteristics of the dredger fill.

Specific Gravity	Water Content /%	Liquid Limit /%	Plastic Limit /%	Plasticity Index	Density /(g/cm^3^)	Organic Matter /%
2.25	253.31	57.03	12.70	44.33	1.83	0.5

**Table 2:** Physical properties of polypropylene fibers.

Length /mm	Diameter /mm	Density /(g/cm^3^)	Tensile Strength /MPa	Fracture Extension /%	Elastic Modulus /MPa
6	0.15	0.91	≥460	≥10	≥3500

**Table 3:** Compositions (wt.%) of cement.

SiO_2_	CaO	Fe_2_O_3_	Al_2_O_3_	Na_2_O	K_2_O	MgO	SO_3_	Others	Loss on Ignition
21.7	57.4	2.9	7.5	0.5	0.4	1.7	3.5	/	4.4

**Table 4:** Testing cases for direct shear test.

W/%	C/%	F/%	T/d	E
0.5 W_L_	0	0, 0.6	0	-
0.5 W_L_	6	0, 0.6, 0.9, 1.2	7	BC
0.5 W_L_	6, 9, 12	0.6	7	WC, SC
0.5 W_L_	6	0.6, 0.9, 1.2	7, 14, 28	WC, SC
0.75 W_L_	6, 9, 12	0.9	7	WC, SC
1.0 W_L_	6, 9, 12	0.9	7	WC, SC
1.5 W_L_	6, 9, 12	0.9	7	WC, SC

Note: W represents the water content; C denotes the cement content; F represents the polypropylene fiber content; T is the curing time; E represents the curing environment; BC represents the standard curing environment; WC represents curing under a freshwater environment; SC represents curing in seawater.

**Table 5:** Value-added contribution and total average contribution of each influencing factor on cohesion in the freshwater curing environment.

Variables in the Model	Contribution	Value-Added Contribution			
		X_1_	X_2_	X_3_	X_4_
When K = 0, the average contribution	0	0.234	0.011	0.079	0.388
X_1_	0.243	-	0.013	0.368	0.204
X_2_	0.011	0.245	-	0.073	0.378
X_3_	0.079	0.532	0.005	-	0.566
X_4_	0.388	0.059	0.001	0.257	-
When K = 1, the average contribution		0.279	0.006	0.233	0.383
X_1_X_2_	0.256	-	-	0.356	0.193
X_1_X_3_	0.611	-	0.001	-	0.286
X_1_X_4_	0.447	-	0.002	0.450	-
X_2_X_3_	0.084	0.528	-	-	0.565
X_2_X_4_	0.389	0.060	-	0.260	-
X_3_X_4_	0.645	0.252	0.004	-	-
When K = 2, the average contribution		0.280	0.002	0.355	0.348
X_1_X_2_X_3_	0.612	-	-	-	0.288
X_1_X_2_X_4_	0.449	-	-	0.451	-
X_1_X_3_X_4_	0.897	-	0.003	-	-
X_2_X_3_X_4_	0.649	0.251	-	-	-
When K = 3, the average contribution		0.251	0.003	0.451	0.288
X_1_X_2_X_3_X_4_	0.900	-	-	-	-
Total average contribution		0.263	0.006	0.280	0.352
Percentage		29.24%	0.63%	31.06%	39.07%

**Table 6:** Value-added contribution and total average contribution of each influencing factor on cohesion in the seawater curing environment.

Variables in the Model	Contribution	Value-Added Contribution			
		X_1_	X_2_	X_3_	X_4_
When K = 0, the average contribution	0	0.270	0.012	0.066	0.379
X_1_	0.270	-	0.015	0.355	0.186
X_2_	0.012	0.273	-	0.060	0.368
X_3_	0.066	0.559	0.006	-	0.543
X_4_	0.379	0.077	0.001	0.230	-
When K = 1, the average contribution		0.303	0.007	0.215	0.366
X_1_X_2_	0.285	-	-	0.341	0.174
X_1_X_3_	0.625	-	0.001	-	0.262
X_1_X_4_	0.456	-	0.003	0.431	-
X_2_X_3_	0.072	0.554	-	-	0.540
X_2_X_4_	0.380	0.079	-	0.232	-
X_3_X_4_	0.609	0.278	0.003	-	-
When K = 2, the average contribution		0.304	0.002	0.355	0.325
X_1_X_2_X_3_	0.626	-	-	-	0.263
X_1_X_2_X_4_	0.459	-	-	0.430	-
X_1_X_3_X_4_	0.887	-	0.002	-	-
X_2_X_3_X_4_	0.612	0.277	-	-	-
When K = 3, the average contribution		0.277	0.002	0.430	0.263
X_1_X_2_X_3_X_4_	0.899	-	-	-	-
Total average contribution		0.288	0.006	0.261	0.333
Percentage		32.44%	0.67%	29.41%	37.49%

**Table 7:** The phase of the relationship results.

Correlation Coefficient	Coefficient of Determination	Adjusted Coefficient of Determination	Standard Error of Estimate
0.950	0.902	0.877	3.982

**Table 8:** Analysis of variance results.

Model	Sum of Squares	Degrees of Freedom	Mean Square	F-Test Statistic	Significance
Regression	2324.540	4	581.135	36.642	0.001
Residuals	253.759	16	15.860		
Sum	2578.299	20			

**Table 9:** The regression equation coefficient and inspection results.

Model	Partial Regression Coefficient		Standardized Partial Regression Coefficient	Independent Variable Test Statistic	Significance
	Regression Coefficient	Standard Error			
Constant	60.202	5.188		11.604	0.001
W	−0.348	0.054	−0.618	−6.408	0.001
F	−3.469	4.646	0.060	−0.747	0.466
C	4.018	0.469	0.792	8.572	0.001
T	0.920	0.135	0.610	6.820	0.001

**Table 10:** The experimental results and predicted values of the samples in the freshwater environment and their errors.

W	F	C	T	Cohesion			
				Experimental Values /kPa	Predicted Values /kPa	Residuals /kPa	Relative Error
28.52	0.60	6.00	7	77.38	78.75	−1.37	−1.76%
28.52	0.90	6.00	7	80.03	77.70	2.33	2.91%
28.52	1.20	6.00	7	71.55	76.66	−5.11	−7.15%
28.52	0.60	6.00	14	90.10	85.19	4.91	5.45%
28.52	0.90	6.00	14	91.16	84.14	7.02	7.70%
28.52	1.20	6.00	14	84.27	83.10	1.17	1.38%
28.52	0.60	6.00	28	95.40	98.07	−2.67	−2.79%
28.52	0.90	6.00	28	100.17	97.02	3.15	3.14%
28.52	1.20	6.00	28	91.16	95.98	−4.82	−5.29%
28.52	0.60	6.00	7	77.38	78.75	−1.37	−1.76%
43.97	0.60	6.00	7	70.49	73.37	−2.88	−4.08%
55.03	0.60	6.00	7	64.13	69.52	−5.39	−8.40%
42.77	0.90	6.00	7	74.20	72.74	1.46	1.96%
55.03	0.90	6.00	7	67.84	68.48	−0.64	−0.94%
85.85	0.90	6.00	7	60.42	57.75	2.67	4.41%
42.77	0.90	9.00	7	87.98	84.80	3.18	3.62%
55.03	0.90	9.00	7	76.83	80.53	−3.70	−4.82%
85.85	0.90	9.00	7	73.67	69.81	3.86	5.24%
42.77	0.90	12.00	7	99.64	96.85	2.79	2.80%
55.03	0.90	12.00	7	90.01	92.58	−2.57	−2.86%
85.85	0.90	12.00	7	80.03	81.86	−1.83	−2.29%

**Table 11:** The experimental results and predicted values of the samples in the seawater environment and their errors.

W	F	C	T	Cohesion			
				Experimental Values /kPa	Predicted Values /kPa	Residuals /kPa	Relative Error
28.52	0.60	6.00	7	74.20	75.02	−0.82	−1.10%
28.52	0.90	6.00	7	76.32	74.02	2.30	3.01%
28.52	1.20	6.00	7	68.37	73.03	−4.66	−6.82%
28.52	0.60	6.00	14	87.45	81.15	6.30	7.20%
28.52	0.90	6.00	14	89.57	80.16	9.41	10.51%
28.52	1.20	6.00	14	81.09	79.17	1.92	2.37%
28.52	0.60	6.00	28	89.57	93.42	−3.85	−4.30%
28.52	0.90	6.00	28	96.46	92.43	4.03	4.18%
28.52	1.20	6.00	28	87.98	91.44	−3.46	−3.93%
28.52	0.60	6.00	7	74.2	75.02	−0.82	−1.10%
43.97	0.60	6.00	7	68.37	69.89	−1.52	−2.22%
55.03	0.60	6.00	7	59.39	66.22	−6.83	−11.51%
42.77	0.90	6.00	7	70.49	69.30	1.19	1.69%
55.03	0.90	6.00	7	62.01	65.23	−3.22	−5.20%
85.85	0.90	6.00	7	56.13	55.02	1.11	1.98%
42.77	0.90	9.00	7	83.74	80.78	2.96	3.53%
55.03	0.90	9.00	7	73.67	76.72	−3.05	−4.13%
85.85	0.90	9.00	7	68.90	66.50	2.40	3.48%
42.77	0.90	12.00	7	93.81	92.26	1.55	1.65%
55.03	0.90	12.00	7	86.92	88.20	−1.28	−1.47%
85.85	0.90	12.00	7	76.32	77.98	−1.66	−2.18%

**Table 12:** The GM (1,1) grey forecasting precision inspection standard.

Standard	C	P
Good	<0.35	>0.95
Qualified	<0.50	>0.80
Pass with difficulty	<0.65	>0.70
Unqualified	≥0.65	≤0.71

## Data Availability

Not applicable.
